# Enzymatic reaction mechanism of *cis*-aconitate decarboxylase based on the crystal structure of IRG1 from *Bacillus subtilis*

**DOI:** 10.1038/s41598-020-68419-y

**Published:** 2020-07-09

**Authors:** Hye Lin Chun, So Yeon Lee, Sung Hoon Lee, Chang Sup Lee, Hyun Ho Park

**Affiliations:** 1 College of Pharmacy, Chung-Ang University, Seoul, 06974 Republic of Korea; 2 College of Pharmacy and Research Institute of Pharmaceutical Science, Gyeongsang National University, Jinju, 52828 Republic of Korea

**Keywords:** Biochemistry, Microbiology, Structural biology

## Abstract

Itaconate, which is formed by decarboxylation of *cis*-aconitate—an intermediate metabolite in the tricarboxylic acid cycle—has been used as a building block in polymer synthesis and is an important chemical in several biomedical and industrial applications. Itaconate is an immunometabolite with antibacterial, antiviral, immunoregulatory, and tumor-promoting activities. Recent focus has been on the role of itaconate in the field of immunology, with immune-responsive gene 1 (IRG1) being identified as the *cis*-aconitate decarboxylase responsible for itaconate production. We solved the structure of IRG1 from *Bacillus subtilis* (bsIRG1) and showed that IRG1 adopts either a closed or an open conformation; bsIRG1 was in the open form. A1 and A2 loops around the active site are flexible and can control the formation of the open and closed forms of IRG1. An in silico docking simulation showed that only the open form of IRG1 can accommodate the substrate. The most energetically favorable position of *cis*-aconitate in the active site of bsIRG1 involved the localization of C2 and C5 of *cis*-aconitate into the H102 region and H151 region of bsIRG1, respectively. Based on the structural study of bsIRG1, compared with IDS epimerase, and in silico docking simulation, we proposed two tentative enzymatic reaction mechanisms of IRG1, a two-base model and a one-base model.

## Introduction

Small metabolites can play important roles in cell growth and survival through various metabolic pathways, including amino acid biosynthesis and energy production, and regulate the activities of various enzymes^1–4^. Decarboxylation, the chemical reaction that removes a carboxyl group from carboxylic acids and releases CO_2_, is a critical reaction in biological systems and is catalyzed by specific enzymes called decarboxylases^5^. Itaconate, which is an unsaturated dicarboxylic acid produced by the decarboxylation of *cis*-aconitate, is an intermediate metabolite in the tricarboxylic acid cycle and is a striking example of a small metabolite that regulates enzyme activity for cell growth and survival^6–8^. According to early studies on various fungi such as *Aspergillus terreus,* which produces high levels of itaconate, itaconate shows antimicrobial activity against *Pseudomonas indigofera* and *Mycobacterium tuberculosis* by directly inhibiting the activity of enzymes such as isocitrate lyase (ICL) and fructose-6-phosphate 2-kinase^7,9,10^. Besides these antibacterial and immunoregulatory activities, itaconate has been used in the polymer industry to generate various resins and bioactive compounds^4,6,11–14^.

**Figure 1:**
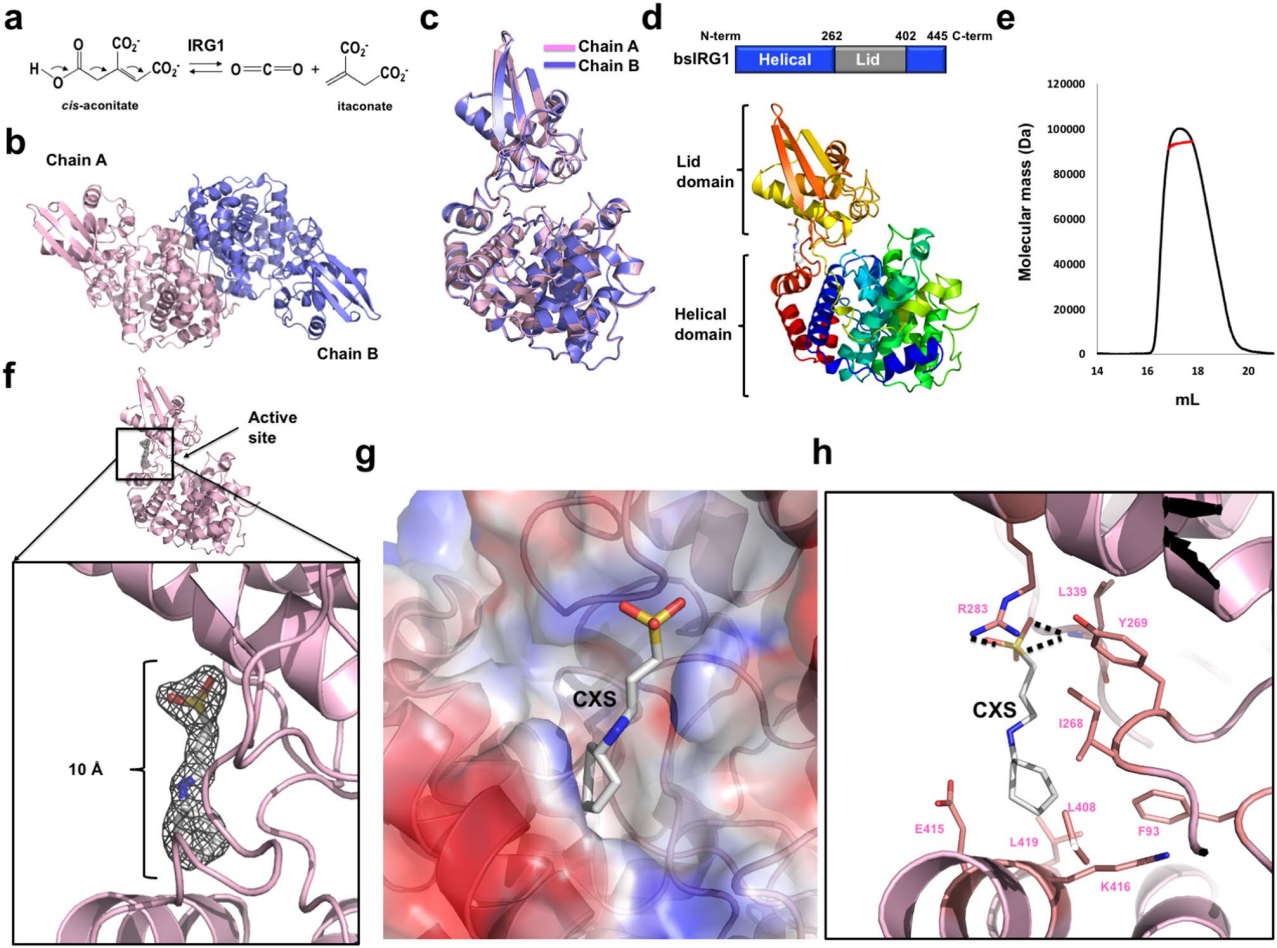
Crystal structure of *Bacillus subtilis* immune-responsive gene 1 (bsIRG1). (**a**) The production of itaconate catalyzed by IRG1 using *cis*-aconitate as a substrate. (**b**) Cartoon representation of dimeric bsIRG1. (**c**) Superposition of chain A and chain B of bsIRG1. (**d**) Domain boundary of bsIRG1. The positions of helical domain and lid domain are shown in the bar diagram shown in the upper panel. The rainbow-colored cartoon representation of monomeric bsIRG1 is shown in the lower panel. The peptide from the N- to C-termini is colored blue to red. (**e**) Multi-angle light scattering profile. Red line indicates the experimental molecular mass. (**f**) Unidentified electron density assigned as 3-cyclohexyl-1-propylsulfonic acid (CXS). 2Fo-Fc density map contoured at the 1σ level around CXS is shown. The magnified view is presented in the lower panel. (**g**) Electrostatic surface representation around the CXS binding pocket of bsIRG1. (**h**) Binding details between CXS and bsIRG1. Amino acid residues from bsIRG1, which are involved in the interaction with CXS, are labeled. Black dashed lines indicate hydrogen bonds.

As part of an effort to identify novel industrial applications of itaconate in polymer science and pharmacy, functional studies of itaconate have been conducted, especially in immune response^12,15^. Initial studies of itaconate production in fungi indicate that the decarboxylation of *cis*-aconitate to generate itaconate was catalyzed by a ~ 55 kDa protein, which was called *cis*-aconitate decarboxylase (CAD)^16,17^. In mammals, high amounts of itaconate are produced by macrophages activated by lipopolysaccharide, which is a major pathogen-associated molecular pattern in gram-negative bacteria^15,18–20^. In a recent study attempting to identify the itaconate-producing enzyme and elucidate the function of itaconate in the mammalian system, immune responsive gene 1 (*IRG1*), which is overexpressed in inflammation following infection by a pathogen, was identified to be gene encoding the enzyme responsible for producing itaconate via decarboxylation of *cis*-aconitate^19^ (Fig. 1a). Antibacterial and antiviral effects of itaconate have been reported in mammals^19,21,22^. During infection with *Salmonella enterica* and *M. tuberculosis*, itaconate has been found to kill these bacteria by targeting ICL, a key enzyme of the glyoxylate shunt^19^. Itaconate also inhibits the activity of succinate dehydrogenase, resulting in a metabolic state that inhibits the replication of the zika virus in infected neurons^21^. *IRG1* overexpression followed by excessive production of itaconate causes gout^23^, chronic arthritis^24^, and tumor progression in mouse models^25^. These findings indicate that IRG1 might be a good target for therapeutic intervention in the diseases mentioned above.

In spite of the increasing interest in the roles of itaconate, the mechanism underlying IRG1-mediated itaconate production using *cis*-aconitate as a substrate remains elusive. In this study, we collected structural information for IRG1 from *Bacillus subtilis* (bsIRG1) and compared it to that for iminodisuccinate (IDS) epimerase, a structural homolog of IRG1; we then performed an in silico docking simulation with the substrate to propose a mechanism for the enzymatic reaction catalyzed by *cis*-aconitate IRG1 to produce itaconate from *cis*-aconitate.

## Results

### Overall structure of IRG1 from *Bacillus subtilis*

At the initial stage of the structural study of the IRG1 family, we selected IRG1 orthologs from three different species, human (NCBI reference sequence ID: NP_001245335.1), mouse (NCBI reference sequence ID: NP_032418.1), and *Bacillus subtilis* (Gene bank ID: ARW33836.1). Although all three genes were overexpressed and their proteins purified, only full-length IRG1 from *B. subtilis* containing residues 1–445 (hereafter called bsIRG1) was crystallized, and the structure was solved. The 1.78 Å crystal structure of full-length bsIRG1 was elucidated by using the molecular replacement (MR) method. The previously reported structural homolog, IDS epimerase (PDB ID: 2HP0) sharing 28% sequence identity with bsIRG1^26^, was used as an initial search model for MR. The structure was refined to R_work_ = 17.7% and R_free_ = 20.8%. The crystallographic and refinement statistics are summarized in Table 1.

**Table 1:** Data collection and refinement statistics.

**Data collection**	
Space group	*P2*_*1*_*2*_*1*_*2*_*1*_
Unit cell parameter	
*a*, *b*, *c* (Å)	a = 9.21, b = 110.87, c = 167.65
α, *β*, γ (°)	α = 90, *β* = 90, γ = 90
Resolution range (Å)^a^	50.00–1.78 (1.82–1.78)
Total reflections	1,297,166
Unique reflections^a^	105,057 (6,857)
Multiplicity	12.3 (11.6)
Completeness (%)^a^	99.8 (99.4)
Mean *I*/σ(*I*)^a^	30.47 (2.07)
*R*_merge_ (%)^a,b^	5.4 (93.3)
Wilson B-factor (Å^2^)	24.5
**Refinement**	
Resolution range (Å)	39.35–1.78
*R*_work_ (%)^a^	17.71 (25.88)
*R*_free_ (%)^a^	20.89 (31.16)
No. of molecules in the asymmetric unit	2
No. of non-hydrogen atoms	7,658
Macromolecules	6,734
Ligands	38
Solvent	886
Average *B*-factor values (Å^2^)	27.0
Ramachandran plot	
Favored/allowed/outliers (%)	98.87/1.13/0
Rotamer outliers (%)	0
Clashscore	2.97
RMSD bonds (Å)/angles (°)	0.007/0.915

*RMSD* root-mean-square deviation.

^a^Values for the outermost resolution shell in parentheses.

^b^R_merge_ = Σ_h_ Σ_i_|*I*(*h*)_i_ −  < *I*(*h*) >|/Σ_*h*_ Σ_*i*_ I(*h*)_*i*_, where *I*(*h*) is the observed intensity of reflection h, and < *I*(*h*) > is the average intensity obtained from multiple measurements.

There were two molecules in the asymmetric unit, chains A and B, both constructed from residues 3 to 445 (Fig. 1b). The extra sequence at the C-terminus (leucine and glutamic acid), which was derived from the plasmid construct, was also included in the final model. The structures of the two chains in the same asymmetric unit were nearly identical, having a root-mean-square deviation (RMSD) of 0.58 Å (Fig. 1c).

The structure of bsIRG1 revealed that it consisted of two distinct domains, a helical domain and a lid domain (Fig. 1d). The helical domain contained 260 residues at the N-terminal and the last 40 residues at the C-terminus, while the lid domain contained approximately 140 residues in the middle of bsIRG1 (Fig. 1d). Because the working stoichiometry of the IRG family has not been studied, and there were two symmetric molecules in the crystallographic asymmetric unit, multi-angle light scattering (MALS) was used to analyze the absolute molecular mass of bsIRG1 in solution. The experimental molecular mass of bsIRG1 in solution determined by MALS was 93.4 kDa (0.48% fitting error). As the calculated molecular weight of the monomeric full-length bsIRG1 (residues 1 to 445), including the C-terminal His-tag, was 47.7 kDa, the peak generated by the dimer indicated that the working stoichiometry of bsIRG1 is that of a dimer in solution (Fig. 1e).

During structural refinement, we observed an unknown electron density on the opposite side of the active site, whose length was around 10 Å (Fig. 1f). Based on this electron density, we searched for a putative molecule using the ligand identification tool in the PHENIX program^27^. This search identified 3-cyclohexyl-1-propylsulfonic acid (CXS) as the molecule that fitted well into the electron density; hence, CXS was included in the model (Fig. 1f). Because CXS was not used for the purification and crystallization steps, endogenous CXS might be incorporated into bsIRG1 in the bacteria during expression. The CXS binding pocket was formed between the lid domain and the helical domain on the opposite side of the active site, and CXS fitted well into the binding pocket (Fig. 1g). The solvent-accessible areas of CXS and bsIRG1 formed by the interaction of CXS with the binding pocket of bsIRG1 were 300 Å^2^ (80% of CXS) and 200 Å^2^ (1.1% of bsIRG1), respectively. The main forces involved in CXS binding to bsIRG1 were hydrogen bonds formed between the sulfonic acid moiety of CXS and Y269, R283, and L339 of bsIRG1, and hydrophobic interactions formed between the cyclohexyl group of CXS and the hydrophobic pocket formed by F93, L408, and L419 of bsIRG1 (Fig. 1h).

### Structural comparison with IDS epimerase, a structural homolog

**Figure 2:**
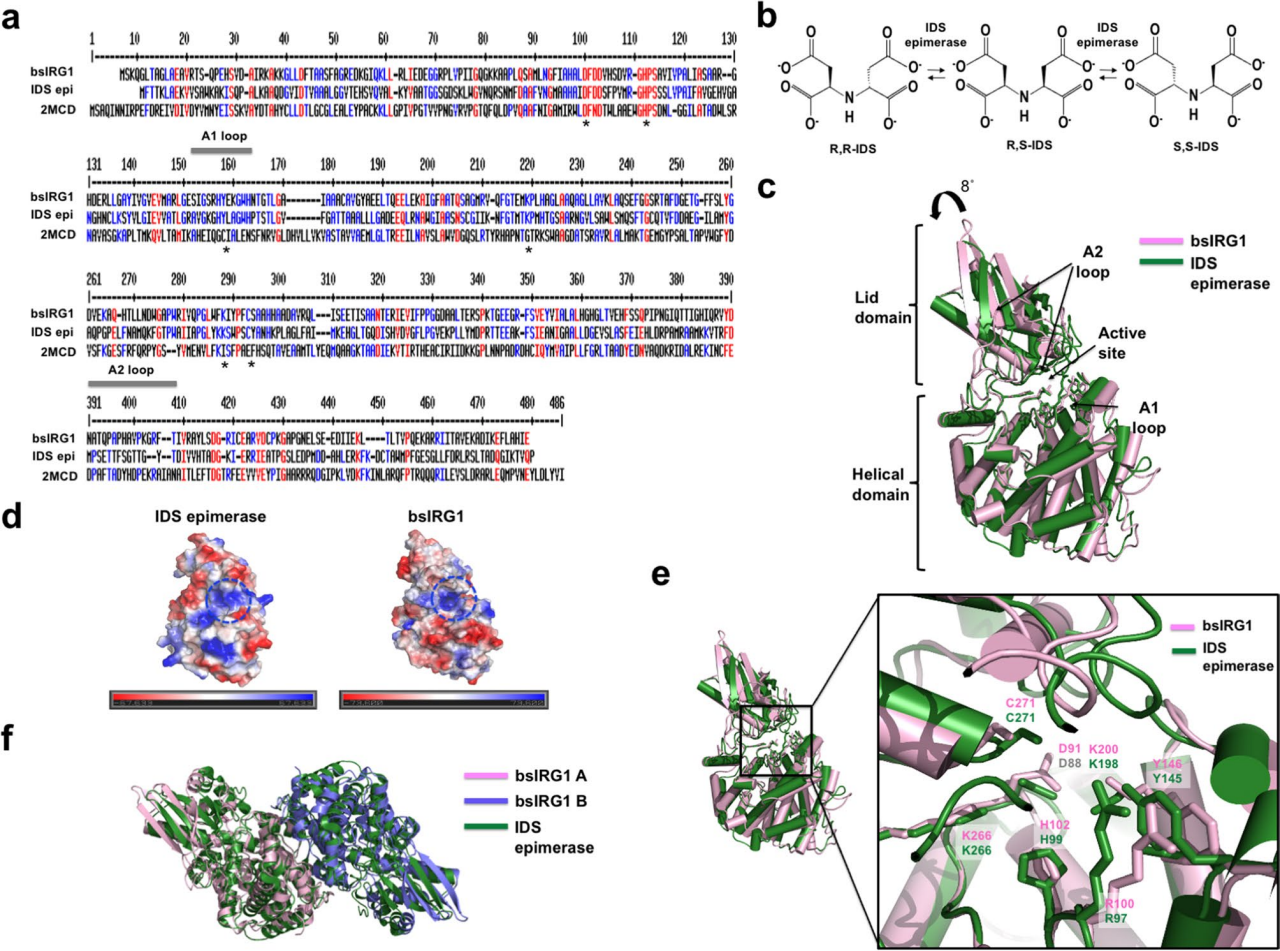
Structural comparison of *Bacillus subtilis* immune-responsive gene 1 (bsIRG1) with iminodisuccinate (IDS) epimerase. (**a**) Sequence alignment of bsIRG1 with IDS epimerase (IDS epi) and 2-methylcitrate dehydratase (2MCD), which are the most similar members of the citrate/2-methylcitrate dehydratase (MmgE)/2-methylcitrate dehydratase (PrpD) protein family. Completely conserved residues and partially conserved residues are indicated in red and blue, respectively. The star (*) symbols indicate the residues conserved in bsIRG1 and IDS epimerase, which are present in the active sites of those enzymes. The positions of the A1 and A2 loops are indicated by the gray bar above the corresponding sequences. (**b**) The production of IDS isomers catalyzed by IDS epimerase. (**c**) Superposition of the structure of bsIRG1 with IDS epimerase. The locations of active site and A1 and A2 loops are indicated. Based on the location of the lid domain of IDS epimerase, the rotation angle of the lid domain of bsIRG1 is also provided above the cartoon. (**d**) Electrostatic surface representation of IDS epimerase and bsIRG1. Blue circles indicate the active sites. (**e**) The details of the active site of bsIRG1 compared to that of IDS epimerase. The close-up view is provided in the right panel. The residues that form the active site are labeled. (**f**) Comparison of dimeric bsIRG1 with dimeric IDS epimerase by superposition analysis.

When we solved the structure of bsIRG1, it was the only structure of IRG family. The structural homolog sharing 28% sequence identity with bsIRG1 was IDS epimerase, a member of the citrate/2-methylcitrate dehydratase (MmgE)/2-methylcitrate dehydratase (PrpD) protein family, that catalyzes the production of the IDS isomer (Fig. 2a)^26^. Among the three different isomers of IDS, R,R isomer, R,S isomer, and S,S isomer, the R,R-isomer and S,S-isomer are converted by IDS epimerase into the R,S-isomer (Fig. 2b)^28^. To understand the working mechanism of IRG1, the structure of bsIRG1 was compared with that of IDS epimerase (PDB ID: 2HP0)^26^. Although the overall structures are similar to each other, both consisting of a helical domain and a lid domain with a root mean square deviation (RMSD) of 2.3 Å, the lid domain of bsIRG1 was tilted by approximately 8° compared to the lid domain of IDS epimerase (Fig. 2c). The most distinct structural difference between the two proteins was the location of the A2 loop, which is one of two loops (A1 loop and A2 loop) located around the active site (Fig. 2c). The A2 loop of bsIRG1 was localized away from the active site, while the A2 loop of IDS epimerase was close to the active site. This indicates that the A2 loop might be flexible in the MmgE/PreD protein family and control the open and closed conformations of this enzyme family (Fig. 2c). The charge distribution and surface features of the two proteins are similar each other, especially in the active site. A deep cavity with basic residues was detected in the active site of both proteins (Fig. 2d). This basic character of the active site could help to accommodate negatively charged carboxyl groups from the substrates, IDS for IDS epimerase and *cis*-aconitate for bsIRG1. The amino acid residues in the active site are completely conserved between IDS epimerase and bsIRG1, although the positions of the side chains of R100 and Y146 were not identical (Fig. 2e).

The dimeric form of IDS epimerase, the functional unit of this enzyme, was also compared with the current dimeric structure of bsIRG1. The structure of the bsIRG1 dimer was almost identical to that of IDS epimerase dimer with an RMSD of 2.5 (Fig. 2f). Both dimeric structures used the same dimerization interface to form a stable dimer (Fig. 2f).

### Structural comparison between IRG1 from different species

**Figure 3:**
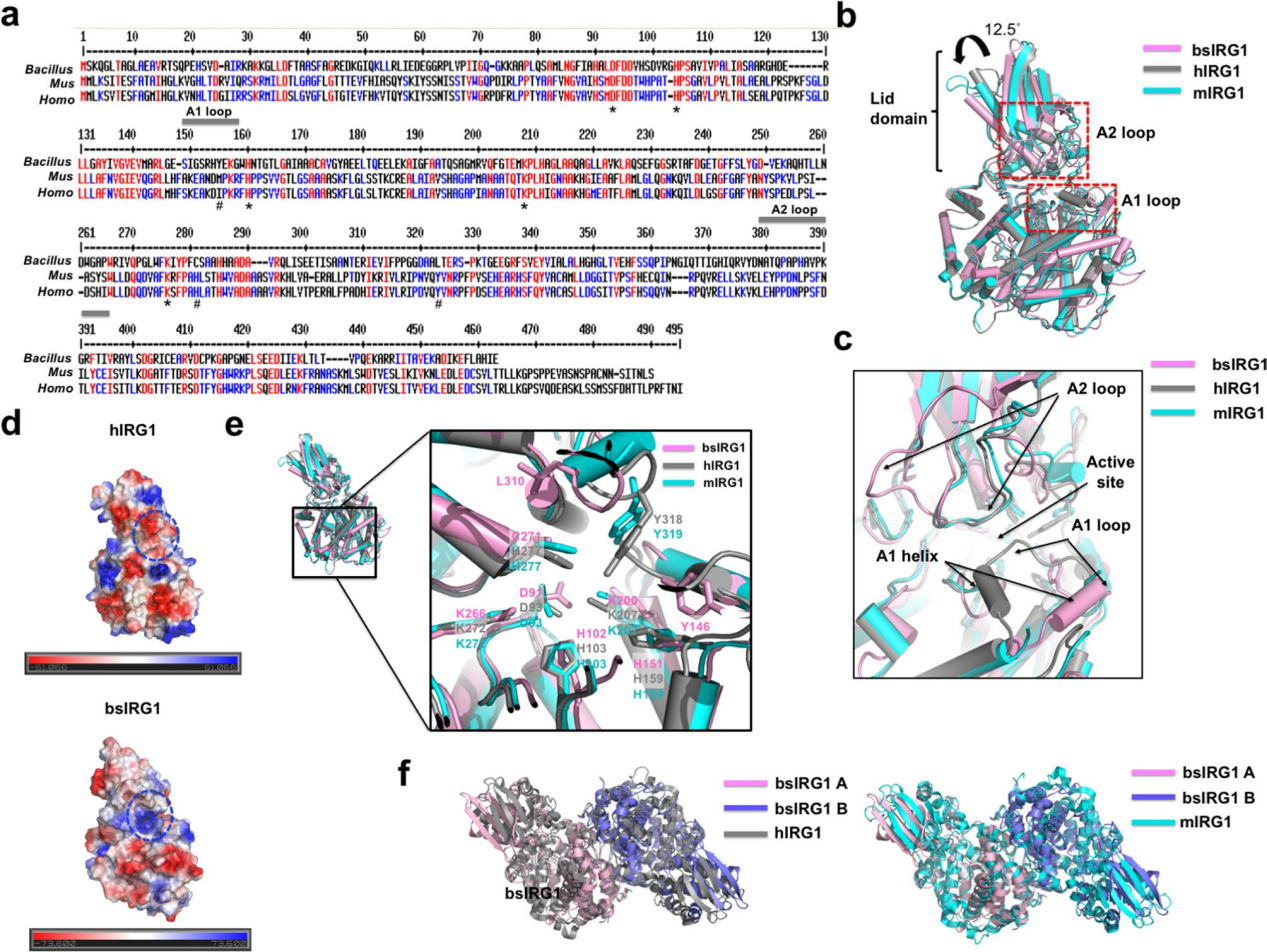
Structural comparison of *Bacillus subtilis* immune-responsive gene 1 (bsIRG1) with mammalian IRG1—hIRG1, and mIRG1. (**a**) Sequence alignment of bsIRG1 with mIRG1 (IRG1 from *Mus musculus*) and hIRG1 (IRG1 from *Homo sapiens*). Completely conserved residues and partially conserved residues are indicated in red and blue, respectively. The star (*) and hash (#) symbols indicate the completely conserved residues and unconserved residues in the active site. The positions of the A1 and A2 loops are indicated by the gray bars above the corresponding sequences. (**b**) Superposition of the structure of bsIRG1 with those of the mammalian IRG1s. The dashed red rectangles indicate the locations of the A1 and A2 loops. Based on the locations of the lid domains of mammalian IRG1s, the rotation angle of the lid domain of bsIRG1 is provided above the cartoon. (**c**) Close-up view focused on the loops around the active site. The locations of the A1 and A2 loops and active site are indicated by black arrows. (**d**) Electrostatic surface representation of hIRG1 and bsIRG1. Blue circles indicate the active sites. (**e**) The details of the active site of bsIRG1 compared to those of mammalian IRG1s. The close-up view is provided in the right panel. The residues that form the active site are labeled. (**f**) Comparison of dimeric bsIRG1 with dimeric mammalian IRGs by superposition analysis.

During the preparation of the manuscript reporting the analysis of the structure of bsIRG1, structures of IRG1 from different species, including mouse (mIRG1) and human (hIRG1), were reported by Pessler and co-workers^29^. bsIRG1 shares sequence identity of 25% with mIRG1 and 24% with hIRG1 (Fig. 3a). To compare the structures of bsIRG1 with those of mIRG1 and hIRG1, the structure of bsIRG1 was superposed on the structures of mIRG1 and hIRG1. Although the overall structure of bsIRG1 was almost identical to those of the other species, with an RMSD of 2.5 Å with mIRG1 and 2.6 Å with hIRG1, the lid domain of bsIRG1 was tilted by around 12.5° (Fig. 3b). Similar to the observation with IDS epimerase, the A2 loop of hIRG1 and mIRG1 showed a closed conformation by its location toward the active site, while the A2 loop of bsIRG1 was wide open in its location away from the active site (Fig. 3b,c). Another distinct structural difference was observed with the connection between the A1 loop and the A1 helix. The structure of this region was located toward the active site with the A2 loop in hIRG1, whereas the same region of bsIRG1 was localized far from the active site (Fig. 3c). Because of the close localization of both the A2 loop and A1 loop connected with the A1 helix around the active site, even covering the active site in the case of hIRG1, the previously reported structure of hIRG1 was considered as a closed form of the IRG1 family. In contrast, the same region of bsIRG1 was dislocated, leaving the active site open, which indicated that our structure might be the open conformation of the IRG1 family. In the case of mIRG1, the model of the A1 loop connected with the A1 helix failed due to the lack of electron density^29^, indicating that this region might be flexible and function as a gate to control the open and closed states.

The closed and open conformations of IRG1 were more distinctly observed in the comparison of surface charge distribution of bsIRG1 and hIRG1. The deep, positively charged cavity that accommodated the negatively charged substrate, *cis*-aconitate, in the active site of bsIRG1 was clearly observed in the surface analysis, while the shallow, negatively charged active site covered by the A2 loop and the A1 loop connected with the A1 helix was observed in hIRG1 (Fig. 3d). These observations indicate that the structure of bsIRG1 is an open form, whereas that of hIRG1 is a closed form. Most of the amino acid residues in the active site are conserved between bsIRG1 and mammalian IRG1 (Fig. 2e). However, unlike the complete conservation of amino acid residues in the active sites of bsIRG1 and IDS epimerase, H277 and Y318 in hIRG1 as well as Y319 in mIRG1—important for the activity of IRG1 in the previous study^29^—are not conserved in bsIRG1 (Fig. 3e).

Because the structure of mammalian IRG1 was dimeric, these dimeric structures were compared with the dimeric structure of bsIRG1 by superposition analysis. The dimeric structure of bsIRG1 was almost identical to that of the mammalian dimer with an RMSD of 2.7 with hIRG1 and 2.8 with mIRG1 (Fig. 3f). All the dimeric structures of IRG1 used the same dimerization interface to form a stable dimer (Fig. 3f).

### Putative catalytic mechanism of itaconate production by IRG1

**Figure 4:**
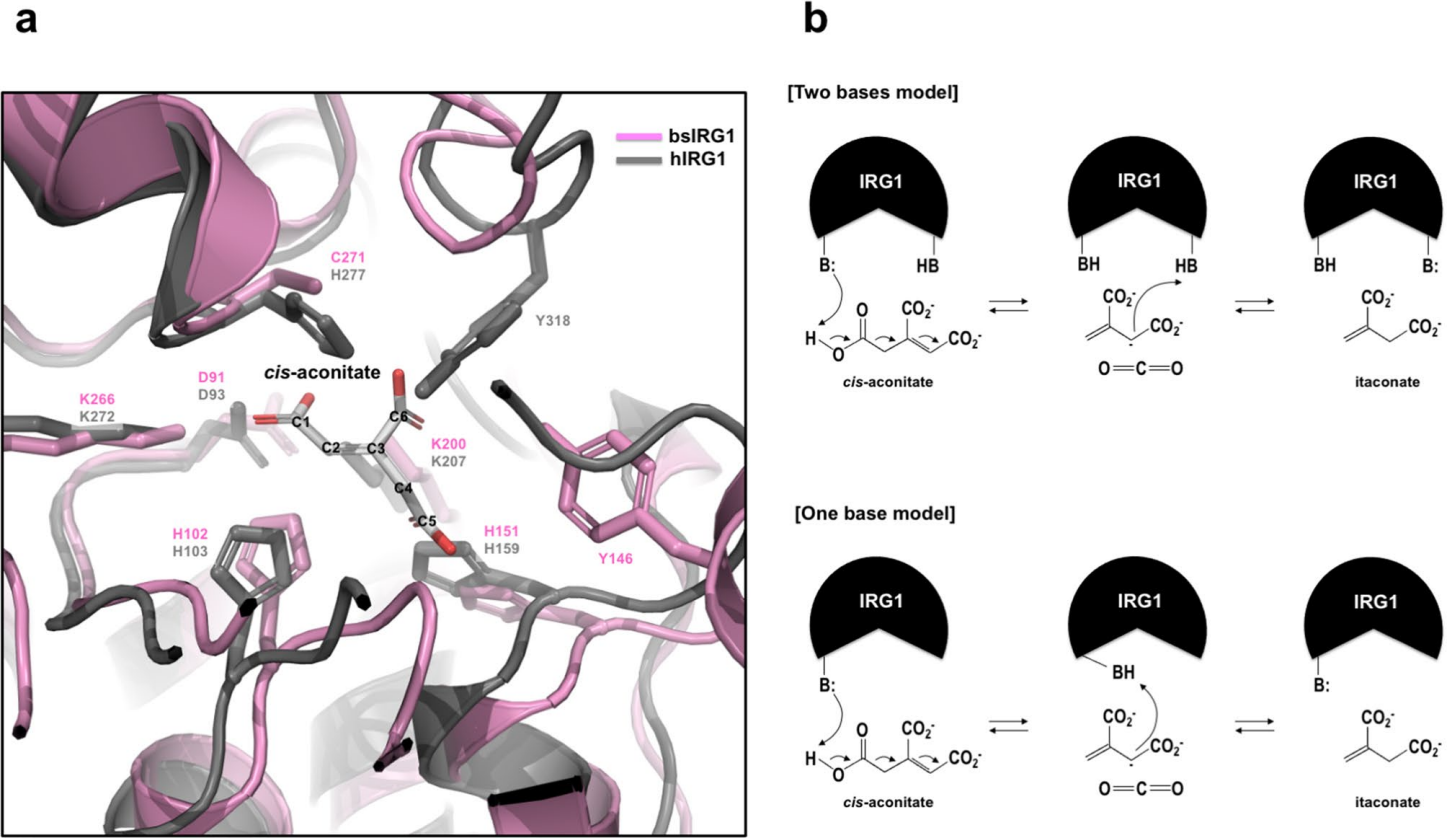
The potential decarboxylation mechanism of immune-responsive gene 1 (IRG1) based on structural and docking simulation analyses. (**a**) Modeling of *cis*-aconitate binding to the active site of *Bacillus subtilis* IRG1 (bsIRG1) and human IRG1 (hIRG1) derived by docking simulation analysis. (**b**) Potential reaction mechanism of IRG1. Two models, two-bases model and one-base model, are proposed.

To understand the catalytic mechanism of IRG1-catalyzed production of itaconate from *cis*-aconitate, we applied our efforts into solving the enzyme/substrate complex structure. However, this effort was not successfully finished; instead, a docking simulation study using MAESTRO docking software was undertaken. An in silico docking simulation of *cis*-aconitate and bsIRG1 indicated that C1 and C6 carboxylate groups of *cis*-aconitate form an extensive network of hydrogen bonds with D91, H102, K200, K266, and C271 from bsIRG1, while C5 carboxylate forms hydrogen bonds only with H151 from bsIRG1 (Fig. 4a). Because it has been reported that C5 carboxylate is a leaving group in the decarboxylation reaction of IRG1^30^, few interactions of the C5 carboxylate with the enzyme will probably facilitate the dissociation of itaconate after the enzymatic reaction of IRG1. In this simulation, Y146, which is not conserved in the mammalian IRG1, was involved in the interaction with the C6 carboxylate. In hIRG1, Y318 performed the role of Y146 of bsIRG1 by interacting with the C6 carboxylate of the substrate (Fig. 4a). H102, which is known to be the most important residue in the IRG1 reaction, was positioned near the C2 region of the substrate where the protonation occurs, indicating that H102 of bsIRG1 (H103 of hIRG1 and mIRG1) might be the basic residue responsible for the protonation and decarboxylation of the substrate (Fig. 4a)^30^.

Based on the structural study of bsIRG1, comparison with IDS epimerase, and the docking simulation, we proposed two tentative enzymatic mechanisms for IRG1. In one mechanism, two bases from the active site of IRG1 are involved in the decarboxylation of *cis*-aconitate. One base facilitates the deprotonation of the C5 carboxyl group, and the other base facilitates the protonation of C2 of the substrate accompanied by the departure of carbon dioxide (CO_2_) (Fig. 4b). This reaction produces itaconate and CO_2_. Another possible mechanism involves only one base from the enzyme, which is involved in both the deprotonation and decarboxylation of *cis*-aconitate (Fig. 4b). In this case, the responsible base residue might be H102 of bsIRG1 (H103 of hIRG1 and mIRG1), which is the most critical residue for the activity of IRG1.

## Discussion

In this study, we solved the structure of IRG1 from *Bacillus subtilis* (bsIRG1) and show that IRG1 can adopt a closed or open conformation, with bsIRG1 being an open form. A1 and A2 loops around the active site are flexible and control the formation of the open and closed forms of IRG1. An in silico docking simulation shows that only the open form of IRG1 can accommodate the substrate. The most energetically favorable position of *cis*-aconitate in the active site of bsIRG1 showed the localization of C2 and C5 of *cis*-aconitate in the H102 region and H151 region of bsIRG1, respectively. Using the structural data of bsIRG1, its comparison with IDS epimerase, and the in silico docking simulation, we proposed two tentative enzymatic reaction mechanisms of IRG1, one involving two bases and the other involving only one base.

Our structural investigation of IRG1 from *B. subtilis* (bsIRG1) shows that bsIRG1 exhibits the typical fold of the MmgE/PrpD family containing two distinct domains, a helical domain and a lid domain. Unexpectedly, unknown electron density was detected on the side opposite to the active site, between the helical domain and the lid domain, and was assigned to 3-cyclohexyl-1-propylsulfonic acid (CXS) by a ligand identification tool. Although the meaning of the occupied CXS is not clear, CXS might help to proper localization of lid domain that might be flexible if CXS is not presented. The lid domain and helical domain is loosely connected by a flexible loop, supporting our speculation of the function of CXS. The more clear function of CXS has to be studied in the future.

Based on the structural comparison of bsIRG1 with IDS epimerase, mIRG1, and hIRG1, we noticed that bsIRG1 was an open form, whose two loops (A1 and A2) were localized to expose the active site. Structural comparison showed that A1 and A2 loops are flexible and control substrate accessibility by working as a gate. The comparison also showed that the lid domain could move to adjust the active site. We also realized that the active site of IRG1, which has decarboxylase activity, and the active site of IDS epimerase, which has epimerization activity, were very similar with respect to the presence of two putative working bases. For IDS epimerase, H99 and Y145 have been suggested as two basic residues for the epimerization reaction^26^. Interestingly, these two residues are conserved only in bsIRG1, but not in mammalian IRG1. In mIRG1 and hIRG1, although H99 is conserved (H103 in mIRG1 and hIRG1), Y145 is not. Unlike the complete conservation of amino acid residues in the active sites of bsIRG1 and IDS epimerase, H277 and Y318 in hIRG1 (Y319 in mIRG1), which were previously shown to be important for the activity of IRG1^29^, are not conserved in bsIRG1. Structural comparison showed that the location of the side chain of Y318 in hIRG1 is similar to that of the side chain of Y146 in bsIRG1, indicating that Y146 of bsIRG1 might share the function of Y318 in hIRG1.

To understand the catalytic mechanism of IRG1, a docking simulation was performed that showed that *cis*-aconitate fit well in the active site of only bsIRG1, but not hIRG1 when the active sites were selected for generating the receptor grid. The inability of the substrate to dock in the active site of hIRG1 might be due to its closed conformation, which did not allow the substrate access to the active site.

Based on the structural study of bsIRG1, comparison with IDS epimerase, and in silico docking simulation, we proposed two tentative enzymatic mechanisms of IRG1, one involving two bases and the other involving one base. In the two-base model, one base facilitates the deprotonation of the C5 carboxyl group, while the other base is involved in the protonation of C2 of the substrate by facilitating the departure of CO_2_ and producing itaconate. Taking into account the position of the side chain, the leaving group (C5 carboxylate), and the place of protonation (C2 region), the bases responsible for the deprotonation of the C5 carboxyl group and protonation of C2 might be H159 and H103, respectively. These two important residues are completely conserved in the different species (Fig. 3a) and are critical for the activity of IRG1^29^. However, if IRG1 were to use only one base for decarboxylation, H103 might be responsible for the protonation and deprotonation processes (Fig. 4b) because this residue was found to be the most important residue for IRG1 activity^29^. Moreover, H99 was the base considered to be critical for the reaction of the same fold protein, IDS epimerase^26^. Thus, H99 of IDS epimerase and H103 of hIRG1 might be the responsible bases for epimerization and decarboxylation, respectively. This base can work alone or work together with another neighboring base. The structure of the substrate/IRG1 complex has to be determined to elucidate the precise working mechanism of IRG1.

## Methods

### Protein expression and purification

The gene for full-length *IRG1* from *Bacillus subtilis* (Gene bank ID: ARW33836.1) was synthesized by BIONICS (Seoul, Republic of Korea) and cloned into a pET21a expression vector. The plasmid encoding the full-length *bsIRG1* was transformed into *Escherichia coli* BL21 (DE3) cells. A single colony was selected and cultured overnight at 37 °C in Lysogeny broth containing 50 µg/mL kanamycin, after which the cells were transferred and cultured in 1 L medium. Isopropyl β-d-1-thiogalactopyranoside (0.5 mM) was added to the medium when the optical density value at 600 nm reached approximately 0.6. The cells were further cultured for 18 h at 20 °C and were harvested by centrifugation at 20 °C. The collected cells were washed with 40 mL of lysis buffer [20 mM Tris–HCl (pH 8.0), 500 mM NaCl, and 25 mM imidazole]. After adding a serine protease inhibitor, phenylmethanesulfonyl fluoride (Sigma-Aldrich, St. Louis, USA), the cells were disrupted by sonication on ice with six bursts of 30 s each and a 90 s interval between two bursts. The lysed cell suspension was centrifuged at 10,000×*g* for 30 min at 4 °C to remove the cell debris. The supernatant was mixed with nickel nitrilotriacetic acid (Ni–NTA) resin (Qiagen, Hilden, Germany) by gentle agitation for 1 h at 4 °C. The resulting mixture was applied to a gravity-flow column pre-equilibrated with lysis buffer. The column was washed with 50 mL of washing buffer [20 mM Tris–HCl (pH 8.0), 500 mM NaCl, and 60 mM imidazole]. Then, a total of 2 mL of elution buffer [20 mM Tris–HCl (pH 7.9), 500 mM NaCl, and 250 mM imidazole] was loaded onto the column to elute the bound protein. The resulting eluate was concentrated to 20 mg/mL and sequentially subjected to size exclusion chromatography (SEC). SEC purification was conducted using an ÄKTA Explorer system (GE Healthcare, Chicago, USA) equipped with a Superdex 200 Increase 10/300 GL 24 mL column (GE Healthcare, Chicago, USA) pre-equilibrated with SEC buffer [20 mM Tris–HCl (pH 8.0), 150 mM NaCl]. Protein fractions were collected, concentrated to 8.3 mg/mL, flash-frozen in liquid N_2_, and stored at − 80 °C until use.

### Crystallization and data collection

For initial crystallization, 1 µL of protein solution was mixed with an equal volume of reservoir solution, and the droplet was allowed to equilibrate against 300 µL of the mother liquor using the hanging drop vapor diffusion method at 20 °C. The initial hit was obtained from a buffer comprising 2.0 M (NH_4_)_2_SO_4_, 0.1 M cacodylate (pH 6.5), and 0.2 M NaCl. The crystallization conditions were further optimized and finally adjusted to a buffer composition of 1.5 M (NH_4_)_2_SO_4_, 0.1 M CAPS (pH 9.8), and 0.2 M Li_2_SO_4_. Qualified crystals appeared in 1 day and grew to a maximum size of 0.2 × 0.5 × 0.2 mm^3^. For data collection, the crystals were soaked in the mother liquor supplemented with 40% (v/v) glycerol as a cryoprotectant and flash-cooled in a stream of N_2_ at − 178 °C. X-ray diffraction data were collected at the Pohang Accelerator Laboratory with the 5C beamline (Pohang, Republic of Korea) at a wavelength of 1.0000 Å. The diffraction data were indexed, integrated, and scaled with the HKL-2000 program^31^.

### Structure determination and analysis

The structure was determined by the molecular replacement (MR) phasing method using PHASER^32^. The previously solved structural homolog IDS epimerase (PDB ID: 2HP0), which shares 28% sequence identity with bsIRG1, was used as a search model^26^. The initial model was built automatically with AutoBuild in PHENIX^27^ and completed with Coot^33^. Model refinement was iteratively performed using phenix.refine in Phenix^27^. The quality of the model was validated using MolProbity^34^. All the structural figures in this paper were generated using the PyMOL program^35^.

### SEC-MALS analysis

The absolute molar mass of bsIRG1 in solution was determined by MALS. The target protein filtered with a 0.2 µm syringe-filter was loaded onto a Superdex 200 10/300 gel-filtration column (GE Healthcare) that had been pre-equilibrated in buffer comprising 20 mM Tris–HCl (pH 8.0) and 150 mM NaCl. The mobile phase buffer flowed at a rate of 0.4 mL/min at room temperature. A DAWN-treos MALS detector (Wyatt Technology, Santa Barbara, USA) was connected with the ÄKTA explorer system (GE Healthcare). The molecular mass of bovine serum albumin was used as a reference value. Data for the absolute molecular mass were assessed using the ASTRA program (Wyatt Technology).

### Sequence alignment

The amino acid sequences of bsIRG1 from different species were analyzed using Clustal Omega (https://www.ebi.ac.uk/Tools/msa/clustalo/).

### In silico molecular docking simulation

In silico docking calculations was performed using GLIDE in MAESTRO program^36^ on a Linux workstation. *cis*-aconitate and bsIRG1 were used as docking ligand and receptor, respectively. At the initial step for the docking process, the ligand (*cis*-aconitate) was prepared by LigPrep module, which performed geometrical refining of chemical structure of *cis*-aconitate and setting up 3D structure with accurate chirality. For the receptor protein preparation, Protein preparation wizard of MAESTRO program was used. Chain A of the solved bsIRG1 structure was selected and edited for missing hydrogens and for assigning proper bond orders. The edited structure was minimized to the default RMSD value. A grid box centered on the residue H102 (H103 in hIRG1) was generated using the default Glide settings. *cis*-aconitate is docked into this defined grid box of the receptor. The constraint to ligand-receptor interaction was not set.

### Structural data accession number

Coordinate and structural factor were deposited in the Protein Data Bank under PDB ID: 7BRA.

## Abbreviations

IRG1: Immune-responsive gene 1
bsIRG1: *Bacillus subtilis* IRG1
hIRG1: Human IRG1
mIRG1: Mouse IRG1
ICL: Isocitrate lyase
CAD: *cis*-Aconitate decarboxylase
MR: Molecular replacement
MALS: Multi-angle light scattering
CXS: 3-Cyclohexyl-1-propylsulfonic acid
IDS epimerase: Iminodisuccinate epimerase
MmgE: Citrate/2-methylcitrate dehydratase
PrpD: 2-Methycitrate dehydratase
RMSD: Root-mean-square deviation
Tris–HCl: Tris(hydroxymethyl)aminomethane hydrochloride
SEC: Size exclusion chromatography
CAPS: *N*-Cyclohexyl-3-aminopropanesulfonic acid
PDB: Protein Data Bank
(NH_4_)_2_SO_4_: Ammonium sulfate
CO_2_: Carbon dioxide
Li_2_SO_4_: Lithium sulfate
NaCl: Sodium chloride
